# Incidence and Predictors of Preeclampsia Among Pregnant Women With Urinary Tract Infection: A Prospective Observational Study

**DOI:** 10.7759/cureus.103840

**Published:** 2026-02-18

**Authors:** Soumya N Doni, Subhashchandra R Mudanur, Rajasri G Yaliwal, Shreedevi Kori, Laxmi Sangolli

**Affiliations:** 1 Obstetrics and Gynaecology, Shri BM Patil Medical College Hospital and Research Centre, BLDE (Deemed to be University), Vijayapura, IND

**Keywords:** antenatal screening, asymptomatic bacteriuria, preeclampsia, pregnancy, urinary tract infection

## Abstract

Introduction: Urinary tract infection (UTI) is one of the most common bacterial infections during pregnancy and has been implicated as a potential risk factor for adverse maternal and perinatal outcomes, including preeclampsia (PE). PE remains a leading cause of maternal and perinatal morbidity and mortality, particularly in developing countries. Increasing evidence suggests that maternal infection and systemic inflammation may contribute to the pathogenesis of PE, warranting further evaluation of the association between UTI during pregnancy and subsequent development of PE. The study aimed to determine the incidence and identify clinical and microbiological predictors of PE among pregnant women diagnosed with UTI.

Materials and methods: This prospective observational study was conducted over 18 months at Shri B.M. Patil Medical College Hospital and Research Centre, BLDE (Deemed to be University), Vijayapura. Pregnant women in any trimester diagnosed with UTI based on clinical features and/or laboratory criteria were enrolled and followed prospectively for the development of PE. Detailed demographic, obstetric, clinical, and laboratory data were collected. UTI was assessed using urine microscopy and culture, while PE was diagnosed according to standard guidelines. Statistical analysis included descriptive statistics, chi-square test for categorical variables, and binary logistic regression to identify predictors of PE within the cohort.

Results: Among 146 pregnant women with UTI, PE developed in 18 (12.3%), representing the incidence of PE within this cohort. Culture-positive infection, higher urine pus cell counts, and UTI diagnosed during the third trimester were significantly associated with the occurrence of PE within the study population (p < 0.05). PE was more frequent among women with increased body mass index, primigravida, and advanced maternal age. On multivariate logistic regression analysis, symptomatic UTI, culture-positive UTI, third-trimester UTI, elevated body mass index, primigravida, and lower educational status emerged as independent predictors of PE.

Conclusion: UTI during pregnancy, particularly when culture positive or occurring in late gestation, was significantly associated with the occurrence of PE within this cohort. However, in the absence of a comparison group without UTI, these findings identify predictors of PE among women with UTI rather than establishing UTI as an independent risk factor. Routine antenatal screening and appropriate management of UTI may help improve maternal monitoring and early identification of hypertensive complications, especially in resource-limited settings.

## Introduction

Urinary tract infection (UTI) remains one of the most common infectious diseases affecting women across all age groups and continues to be a major cause of morbidity and healthcare expenditure worldwide [1,2]. UTI is defined as an inflammatory response of the urothelium to bacterial invasion, usually associated with bacteriuria and pyuria [1]. It accounts for approximately 1-6% of medical referrals to hospitals and may present as either symptomatic or asymptomatic infection [3]. Globally, UTIs and their complications contribute substantially to disease burden, with an estimated 150 million cases reported annually [4,5]. Women are particularly susceptible, with nearly 20% experiencing at least one episode during their lifetime, and prevalence rates of bacteriuria among pregnant women ranging widely from 2% to 41% across different populations [6,7].

Pregnancy predisposes women to UTI due to physiological, anatomical, and immunological changes. Hormonal influences lead to ureteric dilatation, reduced bladder tone, and urinary stasis, facilitating bacterial growth, while hemodilution during pregnancy further lowers urinary antibacterial activity [8]. The risk of UTI begins as early as the sixth week of gestation and peaks between 22 and 24 weeks [9]. The prevalence of asymptomatic bacteriuria (ASB) ranges from 2% to 15% in developing countries and 2% to 7% in developed nations, with higher rates reported from India, likely reflecting socioeconomic factors, poor hygiene, and limited health awareness [10]. If untreated, a UTI during pregnancy may progress to symptomatic infection and is associated with adverse maternal and perinatal outcomes, including preterm labor, low birth weight, maternal anemia, and hypertensive disorders of pregnancy [11].

Preeclampsia (PE) is a multisystem disorder of pregnancy characterized by new-onset hypertension with proteinuria or end-organ dysfunction after 20 weeks of gestation [12]. It affects approximately 2-8% of pregnancies and remains a leading cause of maternal and perinatal morbidity and mortality, particularly in low- and middle-income countries [13]. Despite advances in obstetric care, the pathogenesis of PE remains incompletely understood, although abnormal placentation, endothelial dysfunction, and exaggerated maternal inflammatory responses are central mechanisms [14]. Emerging evidence suggests that infections, including UTI, may amplify systemic inflammation and contribute to the development of PE [14,15]. Several studies and meta-analyses have reported an increased risk of PE among women with UTI, though results remain inconsistent, highlighting the need for further prospective evaluation [15-17]. While some comparative studies have reported an increased occurrence of PE among women with UTI, limited prospective data are available describing the incidence of PE and identifying clinical and microbiological predictors among pregnant women already diagnosed with UTI. Understanding these predictors may help identify high-risk individuals within this population and improve antenatal monitoring and management.

In view of the high prevalence of UTI during pregnancy and the clinical importance of early identification of women at risk of PE, the present study was conducted to prospectively determine the incidence of PE and to identify clinical and microbiological predictors of PE among pregnant women diagnosed with UTI.

## Materials and methods

Study design and setting

This prospective observational cohort study was conducted over a period of 18 months (June 2024 to December 2025) in the Department of Obstetrics and Gynaecology, Shri B.M. Patil Medical College Hospital and Research Centre, BLDE (Deemed to be University), Vijayapura. The study was designed to determine the incidence of PE and to identify clinical and microbiological predictors of PE among pregnant women diagnosed with UTI. Pregnant women attending the antenatal outpatient department (OPD) were screened, and those diagnosed with UTI were enrolled and followed prospectively until delivery for the development of hypertensive disorders of pregnancy. Ethical approval was obtained from the Institutional Ethics Committee (IEC No.: BLDE(DU)/IEC-SBMPMC/064/2023-24).

Inclusion and exclusion criteria

Pregnant women in any trimester diagnosed with UTI based on clinical and/or laboratory criteria were included in the study. The diagnosis of UTI was based on the presence of clinical symptoms such as dysuria, increased frequency of micturition, burning micturition, suprapubic pain, or fever, and/or laboratory evidence of infection, including pyuria and bacteriuria. Women with pre-existing chronic hypertension, diabetes mellitus, chronic renal disease, cardiac disease, liver disease, multiple pregnancy, or those receiving immunosuppressive therapy were excluded from the study to minimize confounding factors that could independently influence the development of PE.

Sample size calculation

The sample size was calculated based on a previous study by Yaqub et al. [18], which reported a mean diastolic blood pressure of 87.85 ± 9.24 mmHg. Considering a 95% confidence level, 5% level of significance (α = 0.05), z value of 1.96, and a margin of error (d) of 1.5, the sample size was calculated using the formula: N = (Z × σ / d​)2, where σ = 9.24. The estimated minimum sample size required was 146.

Clinical and anthropometric assessment

A detailed history and clinical examination were performed for all participants. Obstetric details, including gravidity and gestational age, were recorded. Body mass index (BMI) was calculated using the following formula: weight (kg) divided by height squared (m²). BMI was classified according to the World Health Organization (WHO) criteria as underweight (<18.5 kg/m²), normal (18.5-24.9 kg/m²), overweight/pre-obese (25.0-29.9 kg/m²), obese class I (30.0-34.9 kg/m²), obese class II (35.0-39.9 kg/m²), and obese class III (≥40 kg/m²) [19].

Blood pressure measurement

Blood pressure was measured using a calibrated sphygmomanometer with an appropriately sized cuff after adequate rest. Blood pressure was recorded at enrollment and subsequently monitored at each routine antenatal visit, typically at intervals of four weeks until 28 weeks of gestation, every two weeks until 36 weeks, and weekly thereafter, or more frequently if clinically indicated. Measurements were taken in both sitting and supine positions. Hypertension was defined as systolic blood pressure ≥140 mmHg and/or diastolic blood pressure ≥90 mmHg, recorded on two occasions at least four hours apart, after 20 weeks of gestation, in accordance with the American College of Obstetricians and Gynecologists (ACOG) guidelines [20].

Assessment of proteinuria

Proteinuria was assessed using urine dipstick testing. Significant proteinuria was defined as ≥300 mg protein in a 24-hour urine collection, a protein/creatinine ratio ≥0.3, or a dipstick reading of ≥2+ when quantitative methods were not available, as recommended by the ACOG [20].

Diagnosis and classification of hypertensive disorders of pregnancy

Hypertensive disorders of pregnancy were classified as gestational hypertension, chronic hypertension, preeclampsia, and eclampsia according to the ACOG guidelines [20]. PE was diagnosed as new-onset hypertension after 20 weeks of gestation with proteinuria or, in the absence of proteinuria, evidence of end-organ dysfunction such as thrombocytopenia, renal insufficiency, impaired liver function, pulmonary edema, or new-onset cerebral or visual symptoms [20].

Severity of preeclampsia

PE was further classified based on disease severity in accordance with the ACOG guidelines [20]. PE without severe features (mild PE) was defined as systolic blood pressure ≥140 mmHg but <160 mmHg and/or diastolic blood pressure ≥90 mmHg but <110 mmHg, measured on two occasions at least four hours apart after 20 weeks of gestation, in the presence of proteinuria (≥300 mg/24 hours, protein/creatinine ratio ≥0.3, or dipstick ≥2+), and in the absence of features indicating severe disease [20]. PE with severe features (severe PE) was defined by the presence of any one of the following: systolic blood pressure ≥160 mmHg and/or diastolic blood pressure ≥110 mmHg on two occasions at least four hours apart; thrombocytopenia (platelet count <100,000/µL); impaired liver function (serum transaminases ≥2 times the upper limit of normal) or severe persistent right upper quadrant/epigastric pain; renal insufficiency (serum creatinine >1.1 mg/dL or doubling of baseline creatinine); pulmonary edema; or new-onset cerebral or visual disturbances [20].

Urinary tract infection assessment

Midstream clean-catch urine samples were collected under aseptic precautions. Urine routine microscopy was performed to assess pus cell count. Urine culture and sensitivity testing were conducted using standard microbiological techniques. Significant bacteriuria was defined as ≥10⁵ CFU/mL, and culture positivity and isolated organisms were recorded for analysis [21]. Isolated organisms were recorded for analysis.

Follow-up and outcome measures

All enrolled participants were followed prospectively from the time of diagnosis of UTI until delivery. During follow-up, blood pressure monitoring and urine protein assessment were performed at regular antenatal visits. The primary outcome was the occurrence of PE during pregnancy. The gestational age at onset and severity of PE were documented. This prospective follow-up design allowed temporal assessment of UTI preceding the development of PE within the cohort.

Statistical analysis

Data were entered into Microsoft Excel (Microsoft Corporation, Redmond, WA) and analyzed using SPSS version 26 (IBM Corp., Armonk, NY). Categorical variables were presented as numbers and percentages and were compared using the chi-square test. Binary logistic regression analysis was performed to identify potential clinical and microbiological predictors of PE within the UTI cohort. Given the limited number of outcome events, regression analysis was interpreted as exploratory, and findings were reported cautiously. Odds ratios with 95% confidence intervals were calculated, and a p-value of less than 0.05 was considered statistically significant.

## Results

The study population predominantly comprised women aged ≤30 years (103, 70.5%), with most residing in urban areas (112, 76.7%). A large proportion had attained primary (70, 47.9%) or secondary education (37, 25.3%), while fewer participants were illiterate (22, 15.1%) or college educated (17, 11.6%). The majority belonged to the middle socioeconomic group (87, 59.6%), indicating a largely young, urban, and middle-class cohort (Table 1).

**Table 1:** Socio-demographic characteristics of the study participants. Distribution of the participants according to age group, residence, educational status, and socioeconomic class. Values are presented as frequency (n) and percentage (%).

Variable	Category	Frequency (n)	Percentage (%)
Age (years)	≤30	103	70.5
	>30	43	29.5
Residence	Urban	112	76.7
	Rural	34	23.3
Educational status	Illiterate	22	15.1
	Primary	70	47.9
	Secondary	37	25.3
	College/graduate	17	11.6
Socioeconomic status	Upper middle	38	26.0
	Lower middle	87	59.6
	Upper lower	5	3.4
	Lower	16	11.0

Primigravida women constituted the largest obstetric group (62, 42.5%), followed by gravida 2 (38, 26.0%) and gravida 3 (26, 17.8%), with a progressive decline in higher gravidity. Nearly half of the participants had a normal BMI (72, 49.3%), while a substantial proportion were overweight (46, 31.5%) or obese (10, 6.9%), highlighting a notable burden of increased BMI in the study population (Table 2).

**Table 2:** Obstetric and anthropometric profile of the participants. Distribution of the study participants by gravidity and body mass index (BMI) categories. Values are expressed as frequency (n) and percentage (%).

Variable	Category	Frequency (n)	Percentage (%)
Gravidity	Gravida 1	62	42.5
	Gravida 2	38	26.0
	Gravida 3	26	17.8
	Gravida 4	14	9.6
	Gravida ≥5	6	4.1
Body mass index (kg/m²)	<18.5 (Underweight)	18	12.3
	18.5–24.9 (Normal)	72	49.3
	25.0–29.9 (Overweight)	46	31.5
	30.0–34.9 (Obese I)	8	5.5
	≥35.0 (Obese II & III)	2	1.4

Most participants were symptomatic for UTI, with suprapubic pain being the most frequent symptom (101, 72.9%), followed by increased frequency (48, 32.9%) and burning micturition (47, 30.0%), while 42 (28.8%) women were asymptomatic. Mild pyuria (5-10 pus cells/HPF) was most common (98, 67.1%). Urine culture was positive in 21 (14.4%) cases, predominantly isolating *E. coli* (17, 11.6%), and significant bacteriuria (≥10⁵ CFU/mL) was observed in an equal proportion (21, 14.4%) (Table 3).

**Table 3:** Urinary tract infection (UTI) characteristics. Clinical symptoms, urine microscopy findings, culture results, isolated organisms, and colony counts of urinary tract infection are shown as frequency (n) and percentage (%).

Variable	Category	Frequency (n)	Percentage (%)
UTI symptoms	Asymptomatic	42	28.8
	Burning micturition	47	30.0
	Increased frequency	48	32.9
	Suprapubic pain	101	72.9
Urine pus cells	5–10/HPF	98	67.1
	11–15/HPF	37	25.3
	16–20/HPF	11	7.5
Urine culture	Positive	21	14.4
	Negative	125	85.6
Identified organism	E. coli	17	11.6
	Klebsiella	2	1.4
	Others	2	1.4
Colony count	≥10⁵ CFU/mL	21	14.4
	<10⁵ CFU/mL	125	85.6

Most women were normotensive (128, 87.7%), and new-onset hypertension developed in 18 (12.3%) cases. Proteinuria was absent in the majority (128, 87.7%), with only mild to moderate grades detected in a small proportion. Overall, PE was diagnosed in 18 (12.3%) participants, representing the incidence of PE within this UTI cohort during the follow-up period (Table 4).

**Table 4:** Blood pressure, proteinuria, and preeclampsia status. Classification of the participants based on blood pressure status, new-onset hypertension, degree of proteinuria, and diagnosis of preeclampsia. Values are presented as frequency (n) and percentage (%). Normotensive: SBP <140 mmHg and DBP <90 mmHg. Hypertensive: SBP ≥140 mmHg and/or DBP ≥90 mmHg measured on two occasions. SBP: systolic blood pressure; DBP: diastolic blood pressure.

Variable	Category	Frequency (n)	Percentage (%)
Blood pressure	Normotensive	128	87.7
	Hypertensive	18	12.3
New-onset hypertension	Yes	18	12.3
	No	128	87.7
Proteinuria	None	128	87.7
	1+	8	5.5
	2+	9	6.2
	3+	1	0.7
Preeclampsia (PE)	No	128	87.7
	Yes	18	12.3

Among women with PE, late-onset disease (≥34 weeks) was more frequent (13, 72.2%) compared to early-onset PE (5, 27.8%). Most cases were mild (15, 83.3%), while severe PE was observed in only three (16.7%) women, suggesting predominance of less severe disease (Table 5).

**Table 5:** Clinical profile of preeclampsia (PE) cases (n = 18). Distribution of preeclampsia cases according to gestational age at onset and severity of disease. Values are expressed as frequency (n) and percentage (%).

Variable	Category	Frequency (n)	Percentage (%)
Onset of PE	Early (<34 weeks)	5	27.8
	Late (≥34 weeks)	13	72.2
Severity of PE	Mild	15	83.3
	Severe	3	16.7

Within this cohort of pregnant women with UTI, culture-positive UTI, higher urine pus cell counts (≥8-10/HPF), and UTI diagnosed in the third trimester were significantly associated with the occurrence of PE (Table 6).

**Table 6:** Association of clinical and microbiological characteristics with the occurrence of preeclampsia within the UTI patients. Comparison of urine culture status, urine pus cell count, and trimester of UTI with the occurrence of preeclampsia. Data are presented as numbers and percentages. The chi-square test was used for statistical analysis, with corresponding p-values reported. UTI: urinary tract infection; PE: preeclampsia.

Variable	Category	PE present, n (%)	PE absent, n (%)	Chi-square value	p-value
Urine culture	Positive	11 (7.5)	10 (6.8)	36.403	<0.001
	Negative	7 (4.8)	118 (80.8)		
Urine pus cells	≥8–10/HPF	15 (10.3)	6 (4.1)	79.260	<0.001
	<8/HPF	3 (2.1)	122 (83.6)		
Trimester of UTI	Third trimester	12 (8.2)	46 (31.5)	6.223	0.009
	First/second	6 (4.1)	82 (56.2)		

Maternal age >30 years (11, 7.5%), BMI ≥25 kg/m² (12, 8.2%), and primigravida (13, 8.9%) were significantly associated with the occurrence of PE within the study cohort. Notably, all women who developed PE had new-onset hypertension (18, 12.3%), underscoring its strong association with the disorder (Table 7).

**Table 7:** Maternal factors associated with preeclampsia (PE). Association of maternal age, BMI, gravidity, and new-onset hypertension with preeclampsia status. Values are presented as numbers and percentages. The chi-square test statistics and p-values are shown.

Variable	Category	PE present, n (%)	PE absent, n (%)	Chi-square value	p-value
Age	>30 years	11 (7.5)	42 (28.8)	5.465	0.018
BMI	≥25 kg/m²	12 (8.2)	38 (26.0)	9.583	0.003
Gravidity	Primigravida	13 (8.9)	56 (38.4)	5.132	0.012
New-onset hypertension	Present	18 (12.3)	0 (0.0)	146	<0.001

Binary logistic regression analysis was performed to identify predictors of PE within the UTI cohort. Increased BMI ≥25 kg/m² (OR: 3.40), primary education (OR: 2.50), primigravida status (OR: 2.80), third-trimester UTI (OR: 4.10), and culture-positive UTI (OR: 2.90) were identified as significant predictors of PE occurrence. These findings indicate that both maternal characteristics and specific clinical and microbiological features of UTI were associated with increased occurrence of PE within this cohort (Table 8).

**Table 8:** Multivariable logistic regression analysis of predictors of preeclampsia. Multivariable logistic regression model showing independent predictors of preeclampsia with odds ratios (OR), 95% confidence intervals (CI), and p-values. UTI: urinary tract infection.

Predictor	Odds ratio	95% CI	p-value
Age (>30 years)	1.20	0.99–1.45	0.055
BMI ≥25 kg/m²	3.40	1.60–7.10	<0.01
Primary education	2.50	1.10–5.30	0.03
Primigravida	2.80	1.30–6.00	0.008
History of UTI	1.86	1.61–2.24	0.048
History of hypertension	1.33	1.31–2.14	0.011
Third-trimester UTI	4.10	1.40–12.10	<0.01
Culture-positive UTI	2.90	1.20–6.70	<0.01

## Discussion

PE continues to be a major contributor to maternal and perinatal morbidity and mortality, particularly in developing countries, making identification of modifiable risk factors crucial. Among these, UTI during pregnancy has gained attention as a potential contributor to hypertensive disorders, including PE. Several studies have demonstrated an association between UTI and the development of PE, suggesting that infection-related inflammation may play a role in disease pathogenesis [22,23]. The present prospective cohort study evaluated the incidence and clinical predictors of PE among pregnant women diagnosed with UTI.

The demographic profile of the present study showed that the majority of participants were aged ≤30 years, similar to reports by Zahedkalaei et al. and Arunaa et al., who observed most participants in the reproductive age group of 18-35 years [5,24]. Age was not a distinguishing factor between PE and non-PE groups, in agreement with findings by Izadi et al. [25]. The predominance of urban residents and participants from middle socioeconomic strata in the present study is comparable with earlier Indian studies [24]. Educational status did not show a significant association with the occurrence of PE, consistent with observations by Zahedkalaei et al. [5].

Clinically, primigravida constituted the largest proportion of the study population, which is in agreement with existing literature identifying the first pregnancy as a recognized risk factor for PE [24]. Although nearly half of the participants had a normal BMI, a substantial proportion were overweight or obese. In the present study, increased BMI showed a statistically significant association with PE, supporting reports by Balachandran et al. [26], suggesting that excess maternal weight may contribute to the development of hypertensive disorders in pregnancy.

With respect to UTI characteristics, most participants were symptomatic, while nearly one-third had asymptomatic infection, emphasizing the importance of routine screening during antenatal care. Culture positivity was observed in a minority of cases, with *Escherichia coli* being the predominant uropathogen, consistent with findings by Kaduma et al. [27] and other studies reporting Gram-negative organisms as the most common isolates. Importantly, culture positivity, higher urine pus cell counts, and third-trimester diagnosis of UTI were all significantly associated with the occurrence of PE within this cohort. Similar associations have been reported by Zahedkalaei et al. and Arunaa et al., reinforcing the link between the severity and timing of UTI and the occurrence of PE among affected women [5,24].

Multivariate analysis further strengthened these observations by identifying BMI ≥25 kg/m², primigravida, history of UTI, prior hypertension, third-trimester UTI, and culture-positive UTI as independent predictors of PE. These findings are biologically plausible, as maternal infections may amplify systemic inflammatory responses, leading to endothelial dysfunction, placental ischemia, and the clinical manifestations of PE [28]. Previous studies have similarly implicated UTI in increasing the likelihood and occurrence of PE and related adverse outcomes such as pyelonephritis, prematurity, and low birth weight [11,15]. However, as the present study included only pregnant women with UTI and did not include a comparison group without infection, it cannot determine the relative risk attributable to UTI but rather identifies predictors of PE within this exposed cohort. The temporal pattern of earlier UTI detection followed by later manifestation of PE supports the possible role of infection-related inflammatory pathways in the development of this disorder.

Strengths and limitations

The strengths of the present study include its prospective observational design, which allowed temporal assessment of UTI preceding the development of PE, thereby strengthening the temporal association between exposure and outcome within the cohort. The use of standardized diagnostic criteria for both UTI and PE, along with culture-based confirmation of UTI, enhances the validity and reliability of the findings. Detailed evaluation of clinical, laboratory, and obstetric variables and the application of multivariate logistic regression analysis to identify independent predictors further strengthen the study by minimizing confounding effects. However, certain limitations should be acknowledged. The study was conducted at a single tertiary care center, which may limit the generalizability of the findings to other settings. The moderate sample size may have reduced the power to detect associations for some variables. Additionally, potential confounding factors such as other maternal infections, inflammatory conditions, nutritional status, and prior antibiotic use were not fully assessed. Furthermore, the absence of a comparison group of pregnant women without UTI limits the ability to estimate the relative risk attributable to UTI. As an observational study, a definitive causal relationship between UTI and PE cannot be established, and larger multicentric prospective comparative studies are warranted to confirm these findings.

## Conclusions

This study highlights UTI during pregnancy as an important and potentially modifiable clinical condition associated with the occurrence of PE within this cohort. Women with culture-positive UTI, higher urinary inflammatory burden, and infections occurring later in gestation were more likely to have PE within the study population, supporting the hypothesis that maternal infection may contribute to disease pathogenesis through inflammatory and endothelial mechanisms. The association with maternal characteristics such as increased BMI, primigravida, and lower educational status further emphasizes the multifactorial nature of PE. These findings reinforce the need for vigilant antenatal screening for UTI, including among asymptomatic women, and prompt, effective treatment. However, as this study did not include a comparison group without UTI, these findings should be interpreted as identifying predictors and occurrence of PE within women with UTI rather than establishing UTI as an independent risk factor. Integrating routine UTI surveillance into antenatal care may offer a simple and cost-effective approach to improving maternal monitoring and early detection of hypertensive complications, particularly in resource-constrained settings.
